# Small-cell neuroendocrine carcinoma of the ileum: case report and literature review

**DOI:** 10.1186/s12893-019-0591-8

**Published:** 2019-09-12

**Authors:** Jong Eun Lee, Sung Hoon Hong, Hae Il Jung, Myoung Won Son, Tae Sung Ahn, Sun Wook Han, Jun Hun Cho

**Affiliations:** 1 Department of Surgery, Soonchunhyang Hospital cheonan, College of medicine, 31 Soonchunhyang 6gil, Dongnam-Gu, Cheonan, Chuncheongnam-Do 330-721 South Korea; 2 Department of Pathology, Soonchunhyang Hospital cheonan, College of medicine, Cheonan, South Korea

**Keywords:** Ileum, Neuroendocrine carcinoma, Neuroendocrine tumor

## Abstract

**Background:**

Poorly differentiated neuroendocrine carcinomas (NECs) originating from the gastrointestinal (GI) tract are rare and very highly malignant disease with a poor prognosis. Poorly differentiated NECs most commonly arise in the esophagus and the large bowel; however, they may occur within virtually any portion of the GI tract. It is known, however, that they do not typically occur in the small intestine.

**Case report:**

A 21-year-old woman visited an emergency room with acute abdominal pain that commenced 2 days prior to her presentation. Thereafter, a computed tomography (CT) scan was notable for a small-intestine perforation, and huge masses were observed in the small intestine and the mesentery. The mass that was located at the ileum site is approximately 100 cm above the ileocecal (IC) valve, and while it is located on the anti-mesenteric border and it seems that luminal narrowing had occurred, an obstruction is absent. Also, a same-nature mass is on the mesentery. The pathologic reports confirmed a small-cell-type NEC with a mass size of 7.5 × 6.5 cm. The mitotic count is up to 24/10 high-power fields (HPFs), the results of the immunohistochemical stain are positive for CD56 and synaptophysin, and the Ki-67 level is 50%. %. After the operation, she was treated with Etoposide-Cisplatin (EP) chemotheraphy. Stable disease was seen during Etoposide-Cisplatin chemotheraphy. Liver metastasis was also confirmed after chemotheraphy. Additionally, Irinotecan and cisplatin were used for 3 cycles, but progression of disease, neutropenic fever, thrombocytopenia, general weakness persisted. Eventually, she died 1 year and 6 months after surgery.

**Conclusion:**

Ileum-located NECs are diagnosed very rarely. The most common locations for these tumors along the GI tract are the esophagus and the large intestine, but they can arise anywhere. The prognosis for NECs is poor due to the metastatic disease of most patients at the time of diagnosis. The role of adjuvant treatment requires further evaluation for the attainment of a better understanding of the overall treatment effect.

## Background

Poorly differentiated neuroendocrine carcinomas (NECs) originating from the gastrointestinal (GI) tract are rare and exceptionally harmful illness with a poor prognosis. The knowledge of these malignancies regarding their tumor biology is scant [1]. Poorly differentiated NECs most commonly occur in the esophagus and the large intestine. However, they may occur within almost any portion of the GI tract. It is known, notwithstanding, that they don’t ordinarily happen in the small intestine [2]. Metastasis to other organs has occurred for most patients at the time of diagnosis [3]. The overall survival rate is reported to be extremely poor.

## Case report

**Fig. 1:**
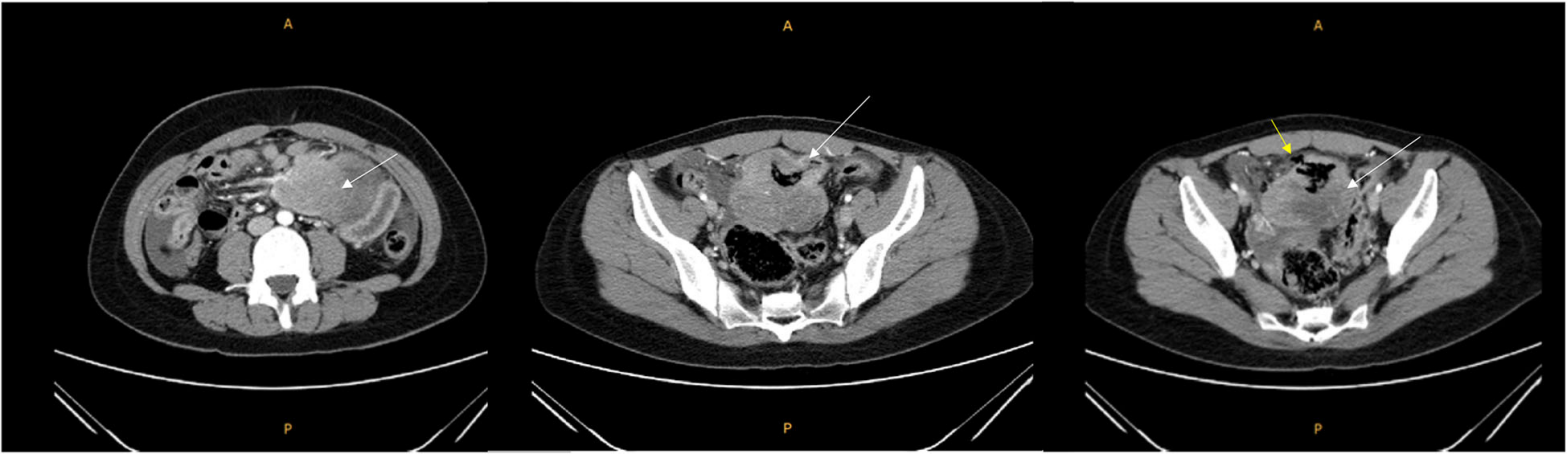
Initial CT scan was notable for multiple huge masses (White arrow: mass, Yellow arrow: perforation site)

**Fig. 2:**
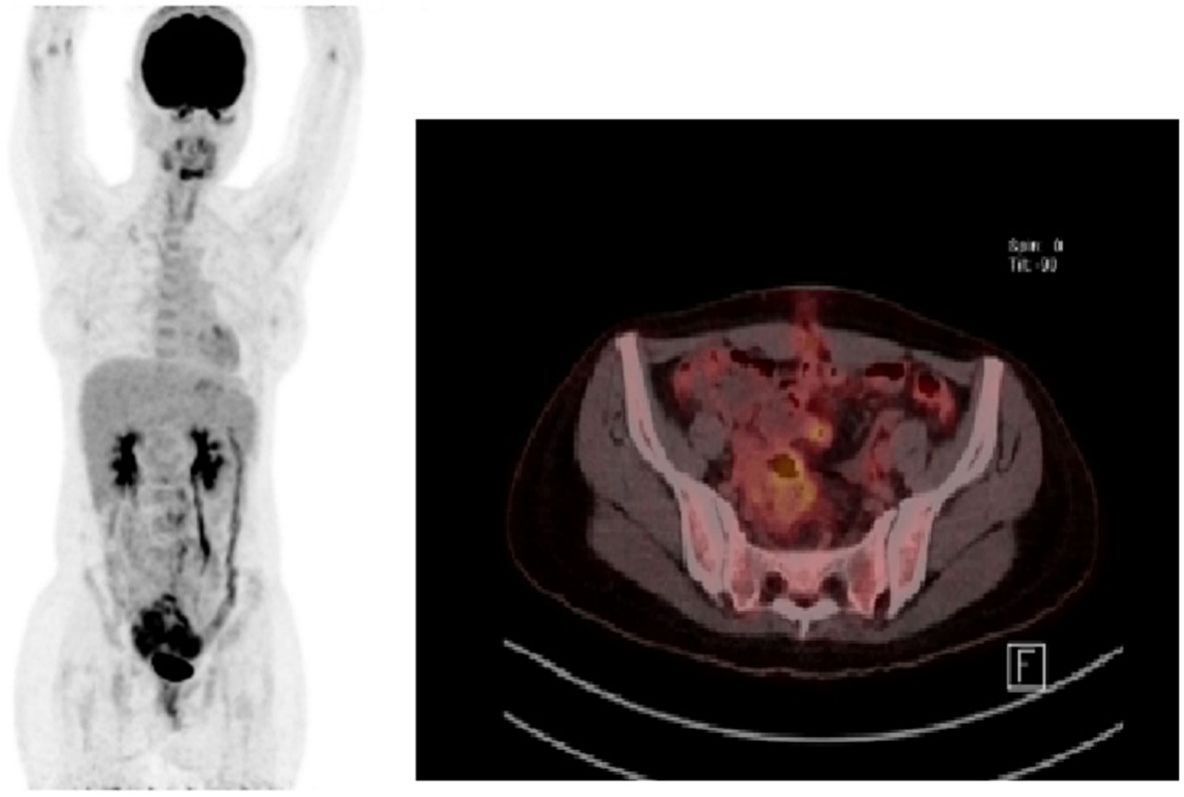
FDG-PET scan show no metastatic lesions in other organ

**Fig. 3:**
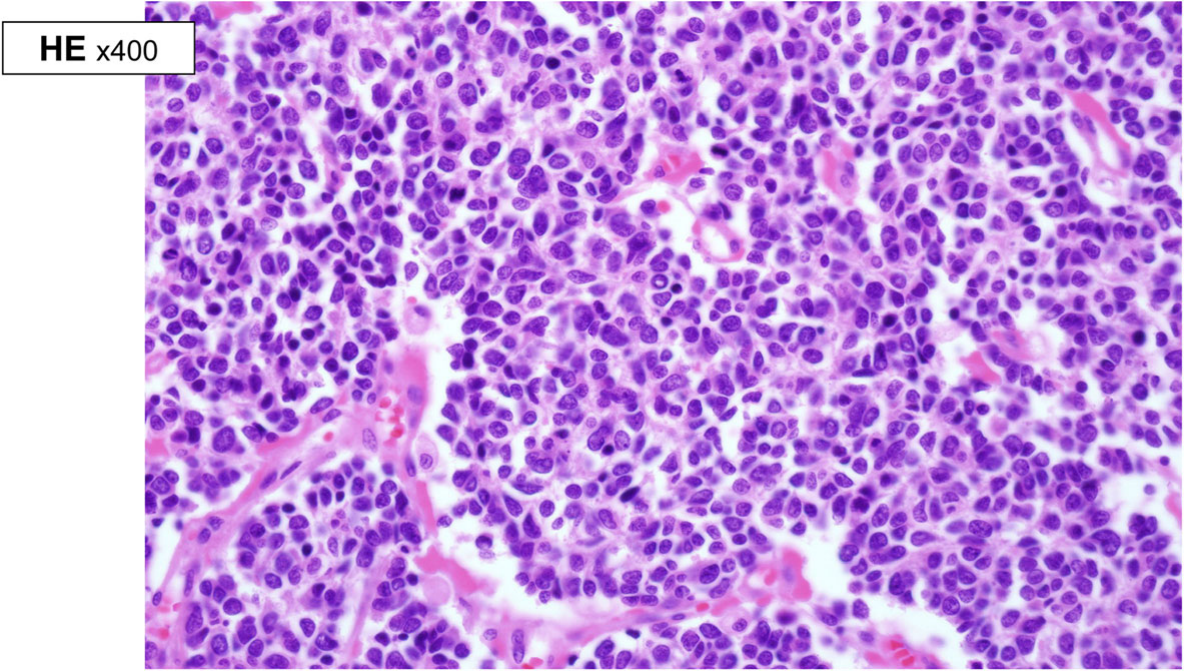
High power view of small cell type neuroendocrine carcinoma with solid pattern. Tumor have monomorphic round, oval or spindle-shaped nuclei and pinkish cytoplasm. Nuclei shows finely stippled chromatin and inconspicuous nucleoli

**Fig. 4:**
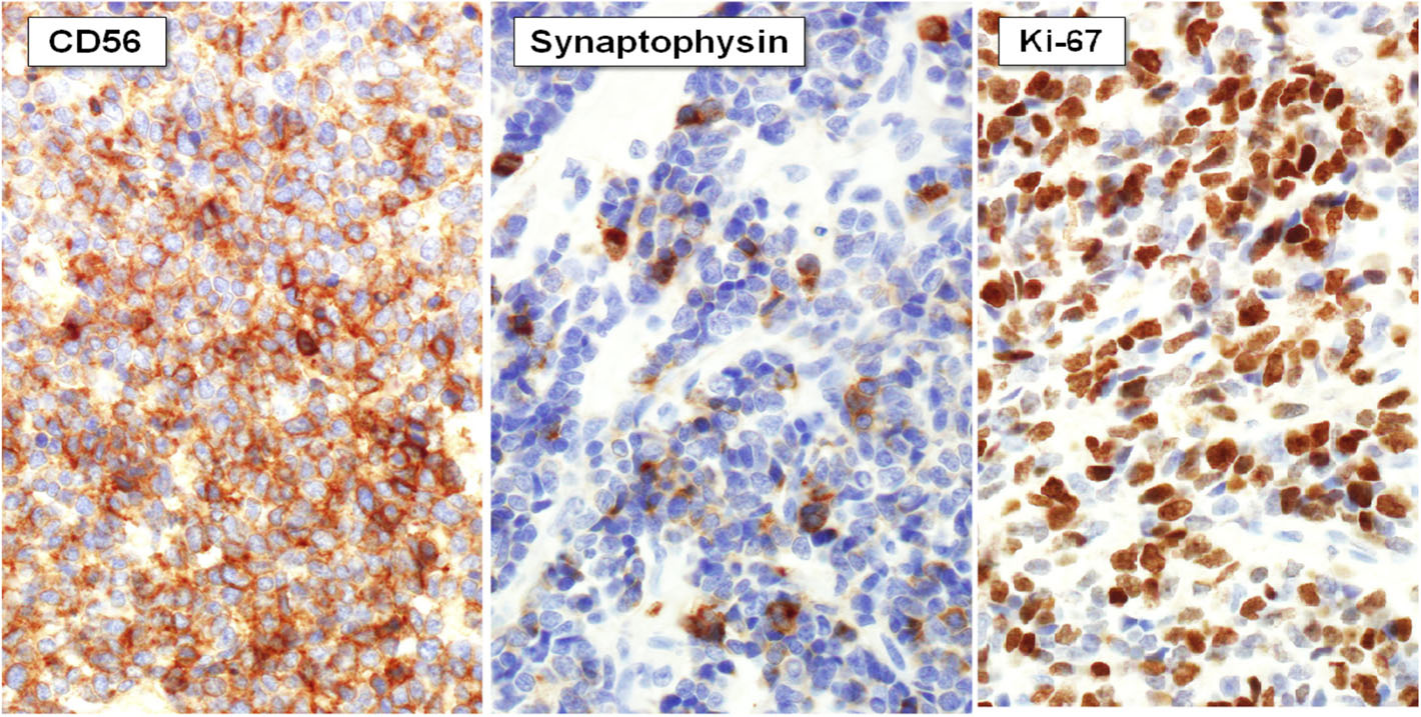
In immunohistochemistry, CD56 was diffusely positive in tumor cells. Synaptophysin showed focal positive staining. Ki-67 labeling index was over than 50%

A 21-year-old woman visited an emergency room with acute abdominal pain that commenced 2 days prior to her presentation. A specific history of the woman was nonexistent, but she had anemia for 1 year for which iron supplements had been taken; it was suspected that the patient had panperitonitis. Thereafter, a computed tomography (CT) scan was notable for a small-intestine perforation, and huge masses were observed in the small intestine and the mesentery. Also, free air was detected around the small intestine and numerous seeding nodules were found in the abdominal cavity, but no other solid-organ metastasis was evident. (Fig. 1) She underwent an emergency operation with a segmental resection of the small intestine and a partial omentectomy. The mass that was located at the ileum site is approximately 100 cm above the ileocecal (IC) valve, and while it is located on the antimesenteric border and it seems that luminal narrowing had occurred, an obstruction is absent. Also, a same-nature mass is on the mesentery. A fluorodeoxyglucose-positron emission tomography (FDG-PET) scan was performed after the surgery to confirm the primary lesions and to identify any additional lesions; no lesions other than those of the abdomen were found. (Fig. 2) The pathologic reports confirmed a small-cell-type NEC with a mass size of 7.5 × 6.5 cm. The mitotic count is up to 24/10 high-power fields (HPFs), the results of the immunohistochemical stain are positive for CD56 and synaptophysin, and the Ki-67 level is over than 50%. (Figs. 3 and 4) After the operation, she was treated with Etoposide-Cisplatin (EP) chemotheraphy. Stable disease was seen during Etoposide-Cisplatin chemotheraphy. However, CT scan was performed after 12 cycles of Etoposide-Cisplatin chemotheraphy, and the intra-abdominal mass was enlarged. In addition, 3 cycles of chemotheraphy with Adriamycin were performed. Liver metastasis was also confirmed after Adriamycin chemotheraphy. For this reason, Irinotecan and cisplatin were used for 3 cycles, but progression of disease, neutropenic fever, thrombocytopenia, general weakness persisted. Eventually, she died 1 year and 6 months after surgery.

## Discussion and conclusions

NECs of the GI tract are uncommon tumors and are classified as either the small-cell or the large-cell type. Thus, NEC of the small bowel is very uncommon. Pathologically, poorly differentiated NECs are similar to small-cell lung cancer (SCLC), and hence their present treatment techniques are similar [4]. NECs are classified with their mitotic count and Ki-67 index. The mitotic counts of poorly differentiated NECs, generally called *high-grade* or *G3 NECs*, are greater than 20 × 10 HPFs, and the Ki-67 level is greater than 20%. The Ki-67 index is most likely the best accessible marker of tumor-cell proliferation. The angioinvasion of high-proliferation tumors with a Ki-67 level higher than 20% is extensive, and these tumors demonstrate an incredible potential to create metastatic disease. As indicated by research effectively detailed that the frequency for positive immunohistochemical reactivity for CK8, synaptophysin, NSE and CD56 in gastrointestinal small-cell NEC was > 90% and that these markers were helpful in diagnosis. Analysis by Shia et al. recognized the following three factors as having an adverse impact on 2-year disease-specific survival: the absence of an adenocarcinoma component (*P* = 0.04), the presence of synaptophysin staining (*P* = 0.05) and progressed disease stage (*P* < 0.0001). The present case has all three of these factors as known already. Subsequently, present case is likewise expected to poor prognosis [5–7]. The G3 NEC is frequently found after the disease has already advanced [8]. Relying upon the location of the lesion in the GI tract, the variety of symptoms like abdominal pain, bleeding [9]. In a number of reports, a distant metastasis was found in more than 50% of the patients at the prognosis stage. Additionally, liver and lymph-node involvement are found in approximately 7–80% of patients at the time of diagnosis [2, 10]. Because the clinical features of patients are very diverse, various treatment modalities are needed and should be applied. Surgery plays an important role not only in the alleviation of symptoms, but also in the pathologic confirmation of the tissue [3]. Surgery is considered to be one of the most important treatment for G1,2 NET without metastasis, whlie there is no definitive evidence that surgery is optimal for G3 NEC. Likewise, For this situation of cutting edge metastatic disease, debulking or cytoreductive surgery and surgical resection of metastatic lesions are not recommended [11, 12]. The adjuvant treatment of the NEC is similar to the SCLC chemotherapeutic agents. The utilization of cisplatin or carboplatin and etoposide for 4–6 cycles is the established treatment for SCLC, and this is additionally utilized for poorly differentiated NECs. With regards to cutting edge SCLC, a randomized control trial conducted in Japan demonstrated that the combination of irinotecan and cisplatin (IP) was related with improved overall survival as compared to the standard cisplatin and EP combination. Two subsequent randomized Western trials, however, failed to confirm this superiority. The two regimens created tantamount adequacy, with less hematological and more prominent GI toxicity with the IP combination [13]. After the 1st line chemotheraphy, most patients eventually relapse and require 2nd line chemotherapy. In general, the prognosis of recurrence and metastasis is very poor, and the reaction to 2nd line chemotherapy will be restricted. As a 2nd line chemotheraphy carboplatin, everolimus, gemcitabine, bevacizumab can be utilized including cisplatin, irinotecan and etoposide. The response rates for these treatments have differed, and further examinations are important to recognize their viability for chemotherapy [14]. Albeit systemic chemotherapy remains the most generally utilized treatment paradigm for these uncommon tumors, restricted information on proper second-line treatment are accessible. Also, sequential radiation can be considered in cases where a higher local-recurrence risk is an issue [15]. In spite of these treatments, however, the disease progression and the metastasis are rapid and frequent, respectively. Recently, a relationship among NEC and the PD-L1 protein was accounted, and an anti-PD-L1 agent has been examined as an NEC treatment. As indicated by certain examinations, the expression of PD-L1 was related with high-grade or G3 NEC subtypes as well as a significantly decreased overall survival. The strong expression of PD-L1 on the NEC tumor cells provides the “adaptive immune resistance” necessary for immune system evasion, which makes the possibility of anti-PD-L1 agents as a new therapeutic modality especially exciting [16, 17]. The other factors like PD-L1 should be developed to provide more appropriate treatment for NEC. Ileum-found NECs are analyzed in all respects seldom. The most common locations for these tumors along the GI tract are the esophagus and the large intestine, however they can emerge anyplace. The prognosis for NECs is poor due to existence of the metastatic lesion at the time of diagnosis. The role of adjuvant chemotherapy requires further evaluation for the attainment of a better understanding of the overall treatment effect. Moreover, different treatment modalities, for example, immunotherapy have been accounted for and further investigation will be required.

## Data Availability

All data generated or analyzed during this study are included in this published article. The data can be obtained by corresponding author.

## Abbreviations

CT: Computed tomography
FDG-PET: Fluorodeoxyglucose-positron emission tomography
HPF: High power field
NEC: Neuroendocrine carcinoma
NSE: Neuron specific enolase
SCLC: Small cell lung cancer
